# Supplementary material for: Eco-Friendly Synthesis of Water-Glass-Based Silica Aerogels via Catechol-Based Modifier
Source: Nanomaterials (Basel). 2020 Dec 1;10(12):2406. doi: 10.3390/nano10122406 (PMC7761200; doi:10.3390/nano10122406)
Supplement: Supplementary file 1 [file nanomaterials-10-02406-s001.pdf]

Supporting information

Eco-Friendly Synthesis of Water-Glass-Based Silica Aerogels via Catechol-Based Modifier

Hyeonjung Kim[1], Kangyong Kim[1], Hyunhong Kim[1], Doo Jin Lee[2] and Jongnam Park[1,3,*]

[1] School of Energy and Chemical Engineering, Ulsan National Institute of Science and Technology (UNIST), Ulsan, 44919, Republic of Korea

[2] DPG Project and Chemical Business Planning Team, SKC, Seoul, 03142, Republic of Korea

[3] Department of Biomedical Engineering, Ulsan National Institute of Science and Technology (UNIST), Ulsan, 44919, Republic of Korea

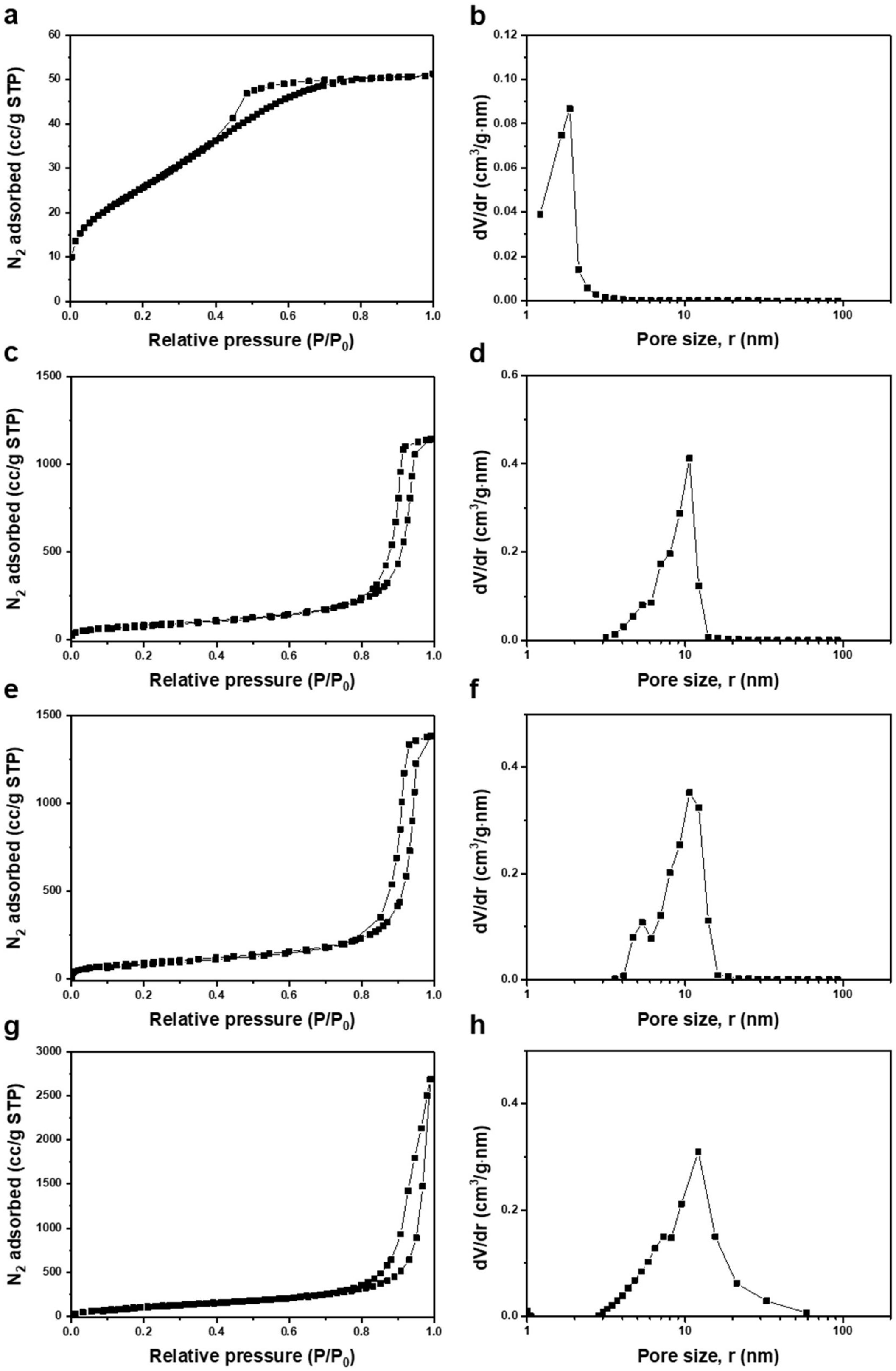

Figure S1. N_2 adsorption-desorption isotherms and the corresponding BJH pore size distributions of (a,b) xerogel, (c,d) TBC, (e,f) TBC+HA, and (g,h) TMCS-modified aerogel.

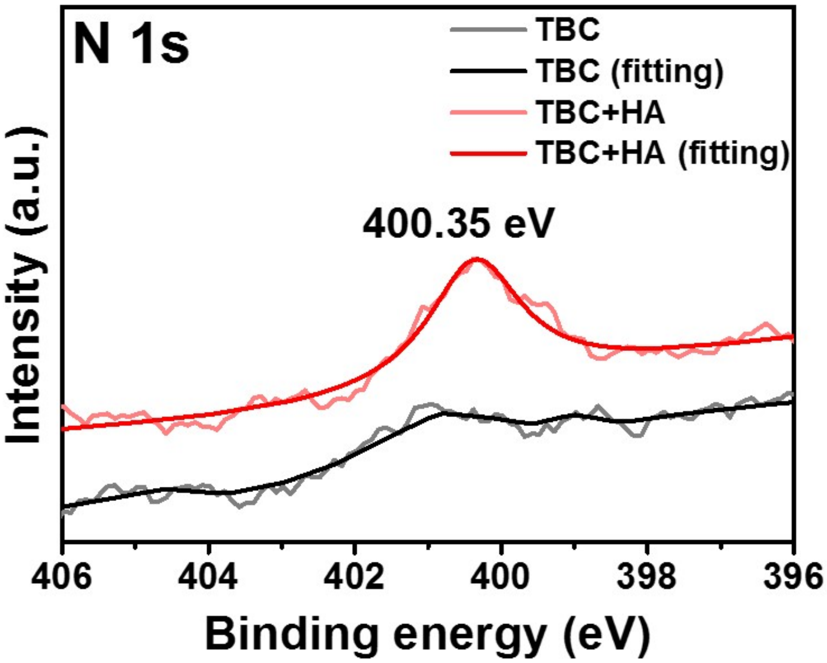

Figure S2. N 1s XPS spectra of TBC and TBC+HA-modified aerogel.

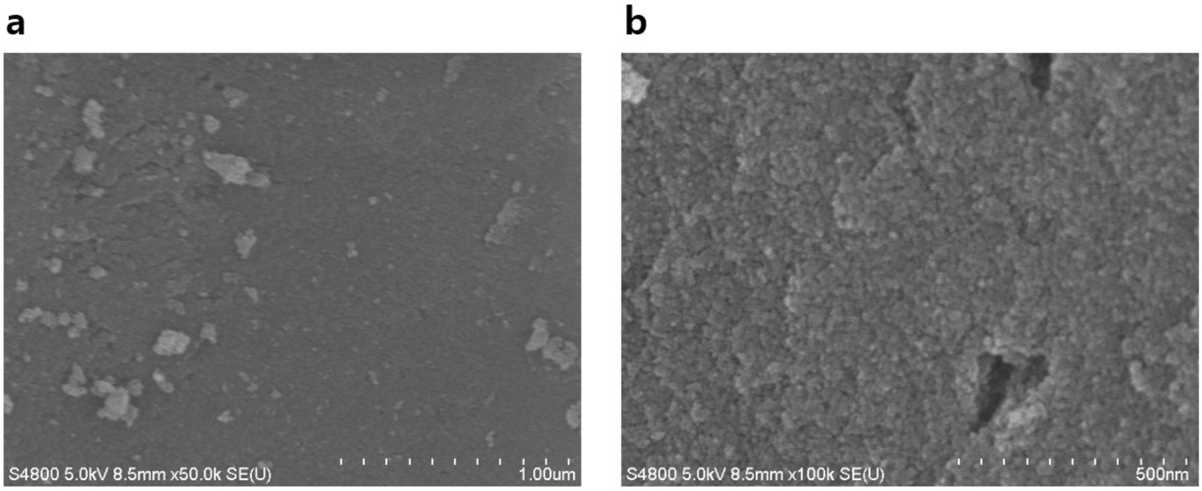

Figure S3. SEM images of PDA+HA-modified aerogel at different magnifications: (a) 50k and (b) 100k.

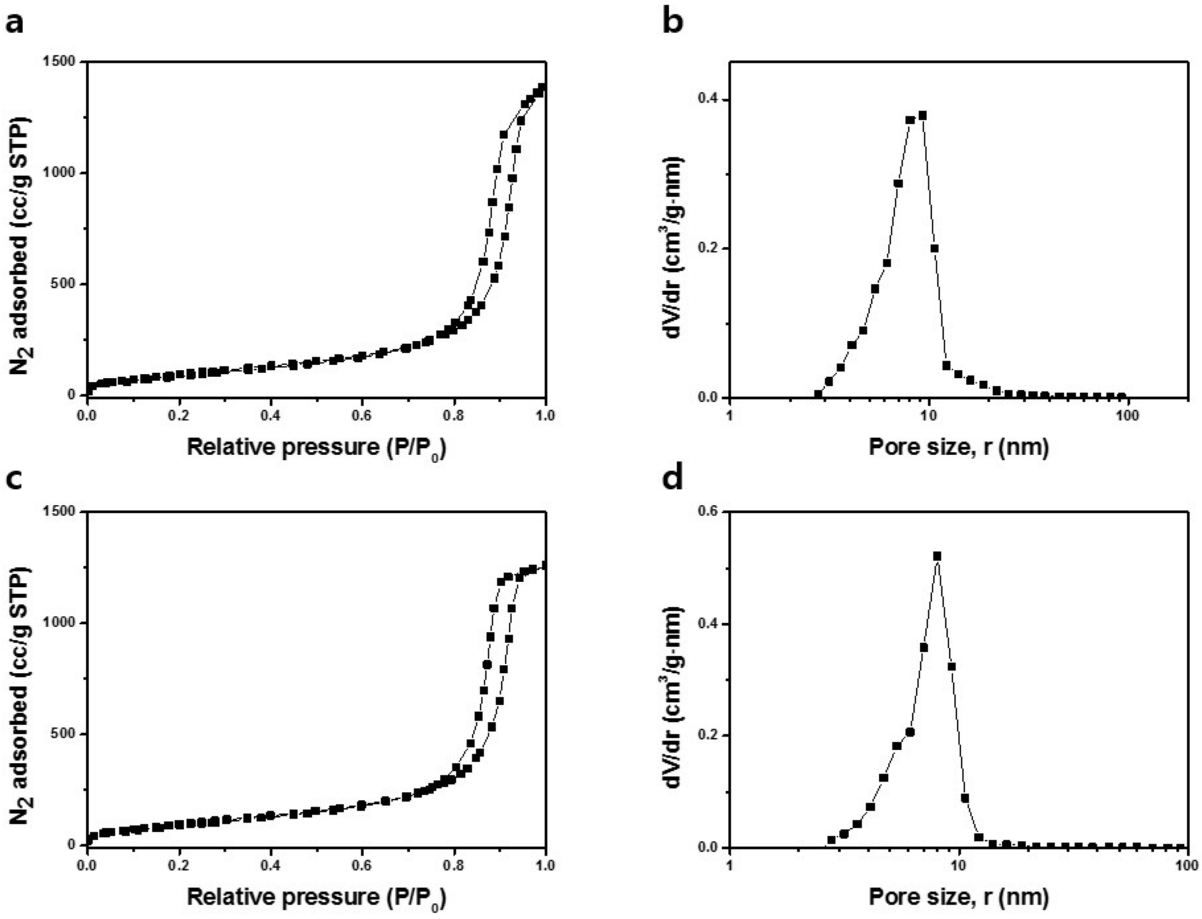

Figure S4. N_2 adsorption-desorption isotherms and the corresponding BJH pore size distributions of (a,b) PDA+TBC and (c,d) PDA+TBC+HA-modified aerogel.

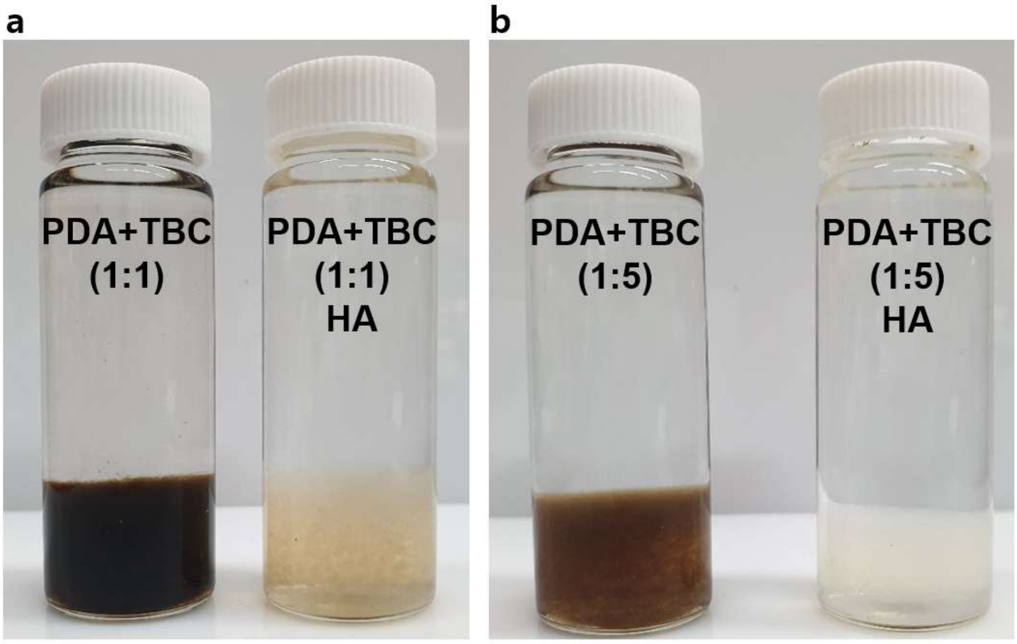

Figure S5. Digital images of various catechol-modified powder aerogels after residual modifier washing steps. Powder aerogels, modified with (a) PDA+TBC (1:1) and PDA+TBC+HA (1:1) and (b) PDA+TBC (1:5) and PDA+TBC+HA (1:5), were soaked in a mixture of ethanol and distilled water.

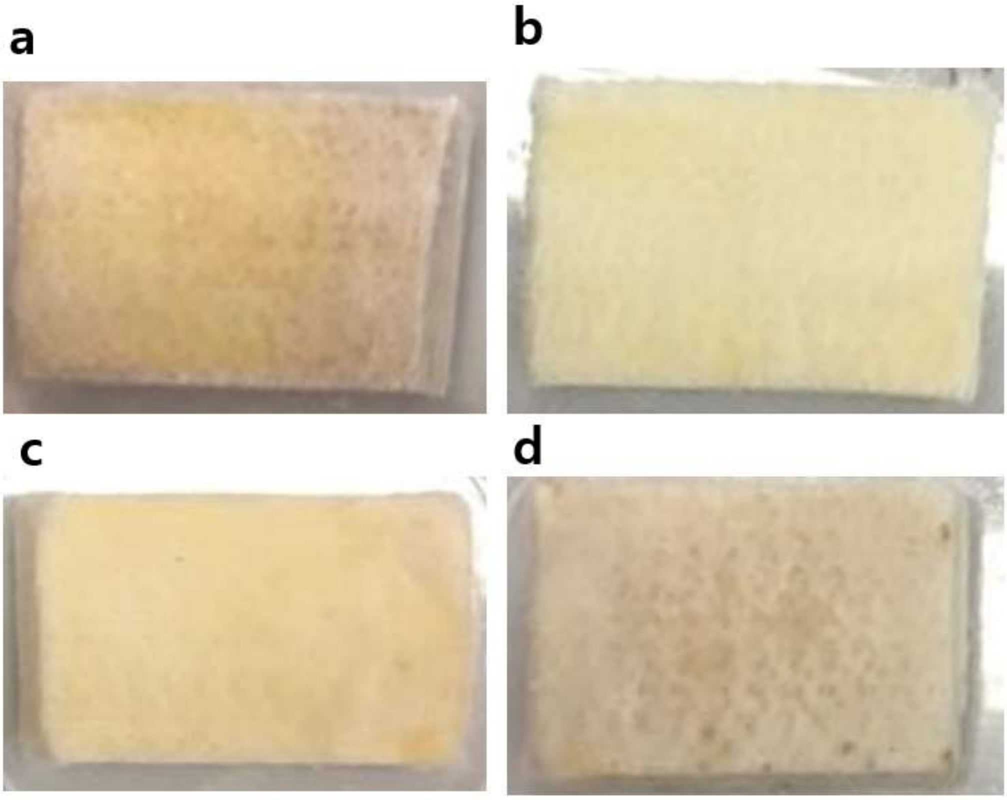

Figure S6. Digital images of glass wool sheets impregnated by (a) xerogel, (b) TBC+HA, (c) PDA+TBC+HA, and (d) TMCS-modified silica aerogel.
